# Socioeconomic inequalities in physical activity among older adults before and during the COVID-19 pandemic: evidence from the English Longitudinal Study of Ageing

**DOI:** 10.1136/bmjph-2023-000100

**Published:** 2023-09-21

**Authors:** Olivia S Malkowski, Nick P Townsend, Mark J Kelson, Charlie E M Foster, Max J Western

**Affiliations:** 1 Department for Health, Centre for Motivation and Health Behaviour Change, University of Bath, Bath, UK; 2 School for Policy Studies, Centre for Exercise, Nutrition and Health Sciences, University of Bristol, Bristol, UK; 3 Department of Mathematics, Institute of Data Science and Artificial Intelligence, University of Exeter, Exeter, UK

**Keywords:** Public Health, Community Health, Epidemiology

## Abstract

**Introduction:**

The influence of the COVID-19 pandemic on physical activity behaviour in older adults is of particular concern. However, little is yet known about how pre-existing socioeconomic inequalities in older adults’ physical activity have been affected by the COVID-19 pandemic. The aim of this study was to explore socioeconomic disparities in physical activity levels and change over time among older adults in England, using data collected before and during the COVID-19 pandemic.

**Methods:**

This longitudinal cohort study analysed data from 3720 older adults (aged 60+ years) who participated in wave 9 (2018/2019) of the main English Longitudinal Study of Ageing (ELSA) survey and wave 2 of the ELSA COVID-19 substudy (November/December 2020). Using multilevel ordinal logistic models, we investigated associations between socioeconomic variables (education, occupational class and wealth) and physical activity, adjusting for potential confounders. We also examined interactions between socioeconomic variables and time (prepandemic vs intrapandemic) to investigate changes in the magnitude of inequalities in physical activity across the two survey periods.

**Results:**

The proportion of participants considered ‘inactive’ rose from 5.7% before the COVID-19 pandemic to 12.5% in November and December 2020. Higher education, occupational class and wealth were positively associated with physical activity before the lockdown. These socioeconomic disparities generally persisted during the COVID-19 pandemic. There was some evidence that differences in physical activity based on education and occupational class reduced during the COVID-19 pandemic, relative to prepandemic data. However, these associations were no longer statistically significant when the three socioeconomic variables and their interactions with time corrected for one another (p>0.05).

**Conclusion:**

Our results suggest there was no additional influence of the COVID-19 pandemic on pre-existing socioeconomic inequalities in older adults’ physical activity levels.

**WHAT IS ALREADY KNOWN ON THIS TOPIC**

**WHAT THIS STUDY ADDS**

- Pre-existing inequalities based on education, occupational class and wealth in older adults’ physical activity largely remained during the COVID-19 pandemic.
- The magnitude of change in physical activity among older adults during the COVID-19 pandemic did not vary according to indicators of socioeconomic status.

**HOW THIS STUDY MIGHT AFFECT RESEARCH, PRACTICE OR POLICY**

- The rising rate of physical inactivity calls for a stepped-up policy response to prevent adverse population health outcomes of the COVID-19 pandemic.
- This study informs practitioners and policymakers about specific subgroups of older adults who may benefit most from interventions to support physical activity.

## Introduction

 The global population of older adults aged 60+ years is projected to double from 1 billion in 2020 to 2.1 billion by 2050.^1^ The health implications of population ageing have commonly been framed in negative terms, with older adults depicted as a social and economic burden.^2^ Yet, substantial interindividual variability exists in the health and functional status of older adults, which is only loosely associated with chronological age.^2 3^ While more work is needed to understand these variations, there is clear evidence of the importance of maintaining healthy lifestyle behaviours in older age, particularly physical activity.^2^ It is well documented that regular physical activity in older adults plays a critical role in the prevention of chronic disease, preservation of physical independence and improvement of quality of life.^4 5^ However, the majority of older adults in England do not meet recommended aerobic or muscle-strengthening physical activity guidelines.^6^

Health disparities in older age are often a consequence of cumulative advantages or disadvantages experienced over an individual’s life course.^7^ Although a variety of measures have been used to characterise socioeconomic status in the literature, some of the most common individual-level indicators include education, occupational class and income/wealth.^8^,^10^ Interestingly, these indicators may be associated with different types of physical activities, suggesting that multiple indicators of socioeconomic status should be considered in physical activity research.^11 12^ Individuals classified as being of higher socioeconomic status (according to diverse indicators of socioeconomic position) consistently report higher physical activity levels than individuals of lower socioeconomic status across the lifespan.^11 12^ This socioeconomic gradient in physical activity participation widens in older age.^13^ Furthermore, research suggests that older adults of lower socioeconomic status experience a greater number of individual and environmental barriers to physical activity than the general older adult population.^14^ Nevertheless, despite being the least active of all adult groups, older adults of low socioeconomic status remain largely absent from the physical activity literature.^6 15^

Since the first recorded case of the SARS-CoV-2 in December 2019, the lives of many people have been disrupted.^16^ Older adults are disproportionately vulnerable to the physiological risks of infection from the coronavirus disease (COVID-19), as well as the psychosocial impacts of distancing and lockdown, such as loneliness and social exclusion.^17 18^ Moreover, lockdown regulations and social isolation during the COVID-19 pandemic have likely contributed to a decline in physical activity among older adults.^19 20^ A recent study, using data from a large representative sample of the English population (n=726 257, aged 16+ years) participating in the Sport England Active Lives Surveys, found that the odds of reporting physical activity were approximately 30% lower during the first national lockdown (April to May 2020), compared with the same time period in previous years; however, the magnitude of these declines differed across sociodemographic groups.^21^

Physical inactivity in older adults is associated with numerous health risks, including more severe COVID-19 outcomes among infected individuals.^22 23^ Therefore, a deeper understanding of changes in older adults’ physical activity levels during the COVID-19 pandemic is warranted.^19 23^ Emerging evidence suggests the COVID-19 pandemic may have exacerbated socioeconomic inequalities in physical activity.^24 25^ While the mechanisms underlying these associations have not yet been explored, it is possible that a range of psychosocial (eg, higher social participation) and environmental factors, known to be important mediators in explaining prepandemic socioeconomic differences in physical activity, may have helped individuals of higher socioeconomic status to maintain healthy lifestyle behaviours during the COVID-19 pandemic.^24^,^26^ Importantly, it remains unclear whether the widening socioeconomic gradients in physical activity participation observed in previous studies conducted during the COVID-19 pandemic are mirrored among the older adult population in England.

The aim of this study was to investigate associations between indicators of socioeconomic status (ie, education, occupational class and wealth), physical activity levels and change over time among older adults in England, using data collected before and during the COVID-19 pandemic. We hypothesised that individuals of higher socioeconomic status would present higher physical activity levels at the prepandemic and intrapandemic assessments, and that socioeconomic inequalities would increase during the COVID-19 pandemic.

## Materials and methods

### Study design and participants

In this study, we used the most recent prepandemic data (wave 9, collected in 2018/2019) from the main English Longitudinal Study of Ageing (ELSA) survey as a baseline assessment,^27^ and data from wave 2 of the ELSA COVID-19 substudy as a follow-up assessment (November/December 2020).^28^ The physical activity items assessed at the first wave (June/July 2020) of the ELSA COVID-19 substudy differed from the other timepoints; data from this wave were therefore not considered in the present study. The sample was limited to core members who participated at both waves of interest, and were aged 60+ years at baseline, to align with WHO’s definition of older age.^1^

ELSA is a longitudinal, biannual survey of adults aged 50+ years living in private households in England. The main survey was established in 2002; the original sample comprised respondents who had participated in the Health Survey for England in 1998, 1999 or 2001. The sample was refreshed periodically to reflect and maintain the complete 50+ years age profile. Further details on the cohort profile are available elsewhere.^29^

The ELSA COVID-19 substudy is a follow-up on select registered participants from the existing ELSA sample in the context of the coronavirus outbreak. Participants in the ELSA COVID-19 substudy were invited to complete the survey online or via computer-assisted telephone interviews. Of the 5378 core members who were successfully interviewed in both wave 9 of ELSA and wave 2 of the ELSA COVID-19 substudy, 4407 were aged 60+ years at baseline. Information about the methods and protocol for the ELSA COVID-19 substudy can be found online.^30^

Procedures were performed in line with national regulations and guidelines for research activities, and all participants provided informed consent. ELSA data from the main survey and COVID-19 substudy are available to access through the UK Data Service (SN 5050 and SN 8688). This study followed the Strengthening the Reporting of Observational Studies in Epidemiology reporting guidelines^31^; the checklist is available online (online supplemental appendix 1).

### Patient and public involvement

Patients or the public were not involved in the design, conduct, reporting or dissemination plans of this research.

### Measures

#### Physical activity

Physical activity data were collected in both waves used for the longitudinal analysis (ie, baseline and wave 2 of the ELSA COVID-19 substudy). Participants were asked to self-report the frequency of their participation in sports or activities that were vigorous, moderately energetic and mildly energetic (more than once a week, once a week, one to three times a month, hardly ever or never). Physical activity was then categorised into four groups in accordance with previous literature: (1) inactive (no activity on a weekly basis); (2) only mild activity at least once per week; (3) at least moderate but no vigorous activity at least once per week; and (4) vigorous activity at least once per week.^32^

#### Socioeconomic variables

Three baseline proxy measures represented socioeconomic status: education, occupational class and wealth.^9^ Education was recoded from six items into three categories: (1) low education (no qualifications); (2) medium education (school qualifications); and (3) high education (at least some higher education). Participants who reported ‘foreign/other’ as their highest educational qualification (~7.6%) were excluded due to their inability to be classified within the educational levels generated for the present study. Occupational class, based on respondents’ current or most recent occupation, was assessed using the three-class National Statistics Socio-Economic Classification.^9^ Participants who had never worked and were long-term unemployed (~0.5%) were excluded from analyses. Wealth was operationalised as total non-pension wealth (quintiles, redefined for the 4407 participants aged 60+ years at baseline) at the benefit unit level.^9^

#### Covariates

Potential confounding variables were chosen based on existing studies. Sociodemographic and health-related covariates retrieved at baseline were biological sex, age (60–69, 70–79 and 80+ years),^33^ ethnicity (dichotomised as white vs non-white in ELSA to avoid disclosure), the number of people in the household (coded as living alone vs not living alone), self-reported limiting long-standing illness, disability, or infirmity (yes/no), and depressive symptoms, assessed with the 8-item Centre for Epidemiologic Studies Depression Scale.^34^ In addition, at the follow-up assessment, respondents were categorised as shielding (yes/no) if they reported staying at home at all times in April 2020.

### Statistical analyses

We defined our complete case sample as participants with complete data on socioeconomic variables and covariates at baseline, and physical activity at both timepoints (prepandemic and intrapandemic). A flow chart depicting the formation of the analytical sample is available in online supplemental figure S1. Analyses were performed using Stata/BE V.17.0 (College Station, Texas: StataCorp). Statistical significance was defined as p≤0.05.

Descriptive statistics were calculated as unweighted frequencies (n) and weighted percentages (%), or weighted mean (SD), using the baseline or follow-up cross-sectional sampling weight as appropriate. The longitudinal ELSA data can be viewed as having a two-level hierarchical structure, with repeated measures (level 1) nested within persons (level 2). Therefore, we constructed a series of multilevel ordered logistic models using the ‘*meologit*’ command, each containing a random intercept at the individual level.

First, we estimated three separate models testing interactions between each of the socioeconomic variables described and time (a binary variable indicating whether the outcome was measured at baseline or during the COVID-19 pandemic), together with their respective constituent main effects (models 1–3). Interaction terms were included to investigate whether the modification effects of each socioeconomic factor varied according to timepoint. In model 4, we fitted a mutually adjusted model with all three socioeconomic variables, time and their interactions considered simultaneously. All models adjusted for covariates. Socioeconomic variables and covariates were treated as time invariant. Analyses were weighted using longitudinal sampling weights to correct for non-response between wave 9 of the main ELSA survey and wave 2 of the ELSA COVID-19 substudy.^30^ Predictive margin probabilities were estimated using the postestimation ‘*margins*’ command for the fixed effects in models 1–3, controlling for the distribution of covariates.

Although single-level tests for the proportional odds assumption are routinely available, these lack applicability to multilevel frameworks. To explore whether the proportional odds assumption was viable for the multilevel models, we fit the underlying series of hierarchical logistic models (models 1–4) ad hoc by creating two dummy variables for the ordinal outcome: (1) inactive versus mild, moderate or vigorous physical activity; and (2) inactive, mild or moderate physical activity versus vigorous physical activity. We then examined departure from consistent patterns of association between the explanatory variables and physical activity. Considering the direction of associations using the binary outcomes was congruous (online supplemental tables S1 and S2), full proportional odds models are presented in this paper. However, we also fit two-level ordinal logistic regression models (models 1–3, unadjusted) using the ‘*gllamm*’ command to relax the proportional odds assumption.^35^ These results are presented in online supplemental tables S3 and S4 for interested readers.

Sensitivity analyses were performed using multiple imputation by chained equations on all variables with missing values under the missing at random assumption. All variables included in analyses were entered as predictors in the imputation model, as well as several auxiliary variables including self-reported general health (1: poor, 5: excellent), alcohol consumption (less than once a week, one to four times per week, five or more times per week) and smoking status (non-smoker vs current smoker). The sample consisted of the 4407 core members who participated in both survey periods of interest. The responses ‘foreign/other’ for participants’ highest educational qualification and ‘never worked and long-term unemployed’ for occupational class were treated as extended missing values and subsequently were not imputed. Twenty imputed data sets were created and combined for analyses using Rubin’s rules. Patterns of missing data are shown in online supplemental table S5. The unadjusted estimates for the complete case and multiple imputation models are presented in online supplemental tables S6 and S7 but are not discussed in the text.

## Results

### Descriptive statistics

The characteristics of the complete case analytical sample are presented in table 1. Of the 3720 included participants, 52.6% were female, 96.4% were white and the mean (SD) age was 70.5 (7.2) years. At baseline, 5.7% of participants were classified as ‘inactive’, compared with 12.5% during the COVID-19 pandemic.

**Table 1:** Participant characteristics

	(n=3720)
Physical activity, n (%)	
Baseline	
Inactive	196 (5.7)
Mild activity	513 (14.1)
Moderate activity	1806 (48.1)
Vigorous activity	1205 (32.1)
During COVID-19	
Inactive	367 (12.5)
Mild activity	503 (15.5)
Moderate activity	1748 (45.5)
Vigorous activity	1102 (26.5)
Education, n (%)	
Low	626 (19.4)
Medium	1390 (38.5)
High	1704 (42.0)
Occupational class, n (%)	
Routine and manual occupations	1199 (35.3)
Intermediate occupations	1034 (27.7)
Higher managerial, administrative and professional occupations	1487 (36.9)
Wealth, n (%)	
1st quintile (lowest)	702 (21.0)
2nd quintile	759 (20.3)
3rd quintile	745 (19.6)
4th quintile	758 (19.7)
5th quintile (highest)	756 (19.5)
Age, mean (SD)*	70.5 (7.2)
Age, n (%)	
60–69 years	1600 (49.9)
70–79 years	1559 (36.5)
80+ years	561 (13.6)
Biological sex, n (%)	
Male	1678 (47.4)
Female	2042 (52.6)
Ethnicity, n (%)	
White	3617 (96.4)
Non-white	103 (3.6)
Living status, n (%)	
Living alone	959 (23.4)
Not living alone	2761 (76.6)
Limiting long-standing illness, disability or infirmity, n (%)	
No	2509 (67.7)
Yes	1211 (32.3)
Depressive symptoms, mean (SD)	1.2 (1.7)
Shielding, n (%)	
No	3271 (85.7)
Yes	449 (14.3)

Unweighted frequencies and weighted percentages are presented. All other values are weighted estimates. All socioeconomic variables and covariates were retrieved at baseline, except for shielding, which was assessed at follow-up. Two participants had missing cross-sectional weight values at follow-up.

* Age was collapsed to 90 for participants aged 90+ years.

n, number of participantsSDstandard deviation

### Associations between socioeconomic variables and physical activity before and during the COVID-19 pandemic

Results for models 1–4 are reported in table 2. In models 1–3, there was a clear gradient in physical activity from highest to lowest education, occupational class and wealth at baseline (all p≤0.01). The odds of engaging in physical activity for participants of high (interaction term: OR=0.64, p=0.006) versus low education (model 1), and in higher (interaction term: OR=0.67, p=0.003) versus routine and manual occupations (model 2), decreased prepandemic to intrapandemic. Baseline associations between socioeconomic variables and physical activity largely remained but were attenuated in the mutually adjusted model (model 4). Patterns of association for interactions between education or occupational class and time were maintained, although statistical significance was lost (all p>0.05). Older participants, respondents of non-white ethnic origin, as well as those reporting a limiting long-standing illness, disability, or infirmity, more depressive symptoms, or shielding during the COVID-19 pandemic had significantly lower odds of physical activity in all tested models (all p≤0.05).

**Table 2:** Multilevel ordered logistic model of physical activity at prepandemic and intrapandemic across socioeconomic groups, adjusted for covariates

	Model 1	Model 2	Model 3	Model 4
**Fixed effects**				
Time				
Baseline (reference)	1.00	1.00	1.00	1.00
During COVID-19	0.95 (0.73, 1.25)	0.89 (0.73, 1.09)	0.88 (0.67, 1.16)	1.09 (0.77, 1.54)
Education				
Low education (reference)	1.00			1.00
Medium education	1.54 (1.18, 2.01)**			1.23 (0.94, 1.61)
High education	3.48 (2.64, 4.59)***			2.06 (1.53, 2.79)***
Occupational class				
Routine and manual occupations (reference)		1.00		1.00
Intermediate occupations		1.90 (1.48, 2.43)***		1.41 (1.09, 1.81)**
Higher occupations		2.76 (2.20, 3.45)***		1.50 (1.16, 1.93)**
Wealth				
1st quintile (reference)			1.00	1.00
2nd quintile			1.82 (1.35, 2.47)***	1.54 (1.14, 2.09)**
3rd quintile			2.20 (1.63, 2.97)***	1.74 (1.29, 2.36)***
4th quintile			3.20 (2.33, 4.40)***	2.24 (1.62, 3.10)***
5th quintile			5.03 (3.61, 7.00)***	3.08 (2.19, 4.33)***
Education×Time*				
Medium versus low	0.76 (0.55, 1.05)			0.82 (0.59, 1.13)
High versus low	0.64 (0.47, 0.88)**			0.74 (0.52, 1.05)
Occupational class×Time*				
Intermediate versus routine and manual		0.77 (0.57, 1.03)		0.82 (0.60, 1.11)
Higher versus routine and manual		0.67 (0.52, 0.87)**		0.75 (0.56, 1.02)
Wealth×Time*				
2nd quintile versus 1st quintile			0.78 (0.54, 1.11)	0.85 (0.60, 1.22)
3rd quintile versus 1st quintile			0.80 (0.55, 1.14)	0.90 (0.62, 1.30)
4th quintile versus 1st quintile			0.73 (0.50, 1.05)	0.88 (0.59, 1.31)
5th quintile versus 1st quintile			0.83 (0.58, 1.19)	1.06 (0.72, 1.56)
Biological sex				
Male (reference)	1.00	1.00	1.00	1.00
Female	0.92 (0.77, 1.09)	0.84 (0.70, 1.00)*	0.82 (0.69, 0.98)*	0.88 (0.74, 1.05)
Age (years)				
60–69 (reference)	1.00	1.00	1.00	1.00
70–79	0.75 (0.62, 0.90)**	0.72 (0.60, 0.86)***	0.69 (0.58, 0.83)***	0.72 (0.60, 0.86)***
80+	0.29 (0.22, 0.37)***	0.25 (0.20, 0.33)***	0.24 (0.19, 0.31)***	0.27 (0.21, 0.35)***
Ethnicity				
White (reference)	1.00	1.00	1.00	1.00
Non-white	0.51 (0.32, 0.82)**	0.54 (0.33, 0.88)**	0.57 (0.35, 0.92)*	0.55 (0.34, 0.90)*
Living status				
Living alone (reference)	1.00	1.00	1.00	1.00
Not living alone	1.23 (1.01, 1.50)*	1.25 (1.02, 1.52)*	0.98 (0.80, 1.20)	1.03 (0.84, 1.26)
Limiting long-standing illness, disability or infirmity				
No (reference)	1.00	1.00	1.00	1.00
Yes	0.24 (0.20, 0.29)***	0.24 (0.20, 0.29)***	0.27 (0.22, 0.32)***	0.27 (0.22, 0.33)***
Depressive symptoms	0.84 (0.80, 0.88)***	0.84 (0.80, 0.88)***	0.85 (0.81, 0.90)***	0.86 (0.82, 0.90)***
Shielding				
No (reference)	1.00	1.00	1.00	1.00
Yes	0.37 (0.29, 0.49)***	0.36 (0.28, 0.48)***	0.39 (0.29, 0.51)***	0.39 (0.30, 0.51)***
**Random effects**				
Variance intercept	2.66 (2.27, 3.12)	2.72 (2.33, 3.18)	2.59 (2.21, 3.04)	2.54 (2.16, 2.98)

Data are odds ratiosORs and 95 % Cconfidence intervalIs. All values are weighted estimates. *p≤0.05, **p≤0.01, ***p≤0.001.

Number of participants=3720 (Llevel 2); number of observations=7440 (Llevel 1).

* Interaction terms.

**Figure 1:**
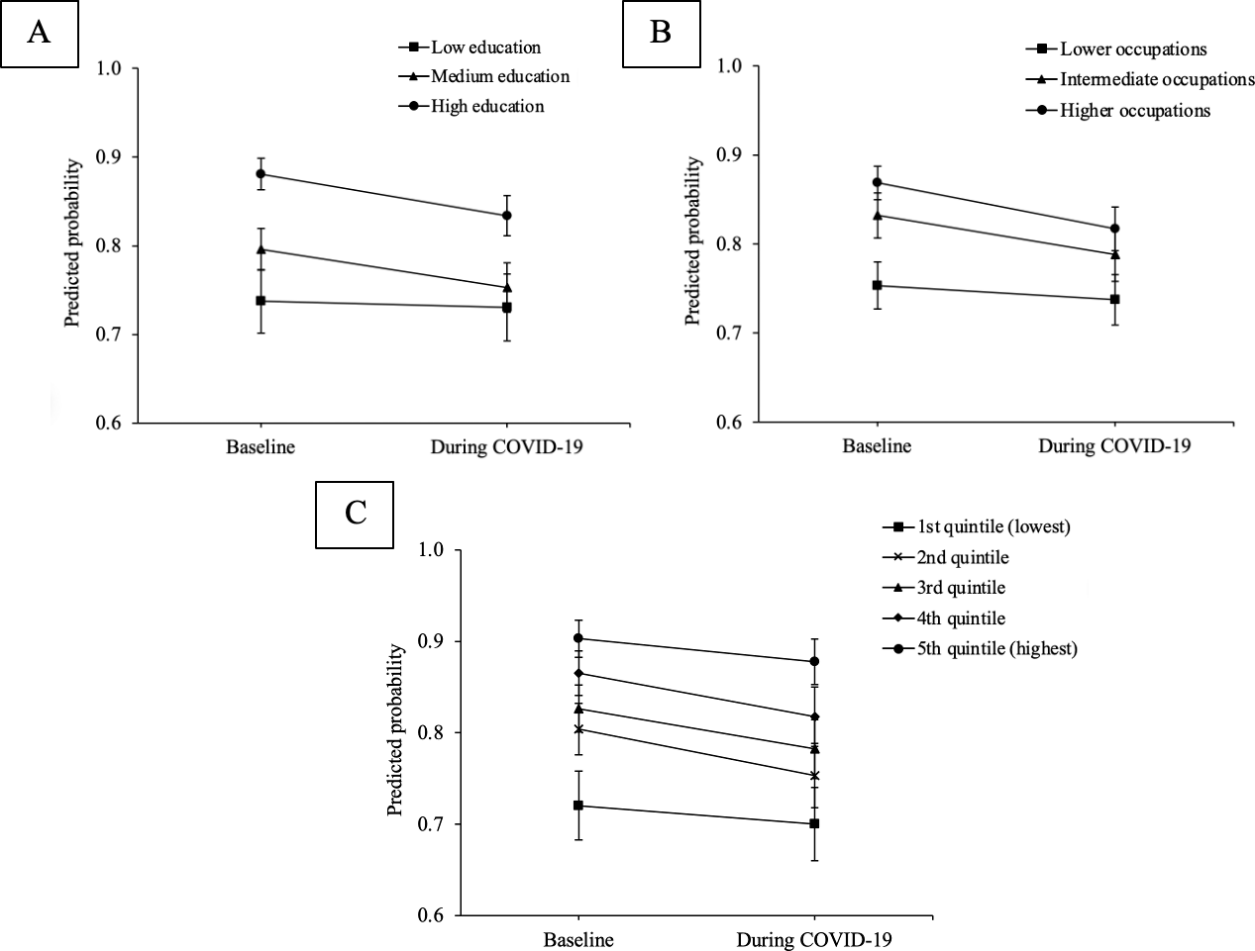
Predicted probabilities of moderate or vigorous physical activity before and during the COVID-19 pandemic by education (A), occupational class (B) and wealth (C). Predicted values were derived from model 1 for education (A), model 2 for occupational class (B) and model 3 for wealth (C). Error bars indicate 95% CIs. Predicted values were computed using the ‘predict (mu fixedonly)’ option to fix the random effects of the respective multilevel ordered logistic model to zero, the ‘asobserved’ option for covariates and the ‘vce(unconditional)’ option.

The predictive margin probabilities (expressed as percentages) across the four levels of the ordinal physical activity outcome variable before and during the COVID-19 pandemic, by socioeconomic groups, are presented in online supplemental table S8. The collapsed (for ease of visualisation) predicted probabilities of engaging in moderate or vigorous physical activity at prepandemic and intrapandemic across socioeconomic groups are represented graphically in figure 1. Participants with higher education, occupations and wealth had greater probabilities of reporting moderate or vigorous physical activity at both survey periods, suggesting that socioeconomic inequalities persisted during the COVID-19 pandemic.

### Sensitivity analyses

When performing multilevel ordinal logistic models with imputed data (online supplemental table S9), results were broadly similar. Some additional interactions that were not observed in the complete case analyses emerged in the separate models (ie, second, third and fourth quintiles vs first quintile of wealth), suggesting the differential in the odds of physical activity between participants in higher quintiles of the wealth distribution, relative to the lowest quintile, reduced during the COVID-19 pandemic. However, these associations were no longer statistically significant in the mutually adjusted model (model 4).

## Discussion

In this study, we investigated associations between socioeconomic variables and physical activity before and during the COVID-19 pandemic (when the second national lockdown restrictions and social distancing regulations were in place in the UK) in a sample of older adults living in England, aged 60+ years. Although there was no statistically significant difference in physical activity levels before versus during the COVID-19 pandemic in the multilevel models, the descriptive statistics suggested a slight decrease over time, with more participants classified as inactive (12.5% vs 5.7%) at follow-up. As hypothesised, we found evidence of pre-existing socioeconomic inequalities in physical activity based on education, occupational class and wealth. Results also suggested that socioeconomic disparities persisted during the COVID-19 pandemic. However, in contrast to our hypothesis, there was inconclusive evidence regarding any socioeconomic differences in physical activity change between the two survey periods. All models controlled for biological sex, age, ethnicity, living status, the presence of any limiting long-standing illness, disability, or infirmity, depressive symptoms, and shielding during the COVID-19 pandemic. The main findings remained unchanged when analyses were performed using multiple imputation.

Our result that older adults with higher education, occupational class and wealth had increased odds of engaging in physical activity before the lockdown agrees with previous work.^11 12^ This study contributes to the extant literature by providing evidence of persistent socioeconomic disparities in older adults’ physical activity levels during the COVID-19 pandemic, a finding that may extend to other age groups.^25^ Although the separate models showed some evidence of decreasing socioeconomic gradients in physical activity based on education and occupational class, these results were not observed in the mutually adjusted model, where socioeconomic variables and their interactions with time corrected for one another. Interestingly, previous studies found that socioeconomic inequalities (based on education, occupational class and income) in physical activity were exacerbated during the COVID-19 pandemic.^25 36^ Nevertheless, these studies used different physical activity measurement instruments and examined associations in samples across the adult age range. It is therefore possible that socioeconomic indicators are less pertinent explanatory variables of physical activity change in older adults. Another plausible explanation is that younger adults were more reliant on the physical activities most affected by the COVID-19 pandemic and showed declines in activity levels due to changes in employment status (eg, furlough) or childcare responsibilities among other factors.^21^ These postulations warrant confirmation in future studies. In addition, age, ethnicity, the presence of any limiting long-standing illness, disability, or infirmity, depressive symptoms, and shielding during the COVID-19 pandemic were important covariates and may be useful candidates for interaction with indicators of time or socioeconomic status in further research.

This study was strengthened by the large, nationally representative sample of older adults in ELSA, as well as the longitudinal design which enabled the comparison of prepandemic and intrapandemic associations. Nevertheless, our findings should be interpreted in light of several limitations. Notably, in longitudinal studies with older adults, considerable attrition occurs due to death, which is only partially corrected by applying weights to statistical analyses.^33 37^ Second, the follow-up assessment for physical activity took place in November/December 2020. However, the starkest changes in physical activity may have occurred earlier in the COVID-19 pandemic during the critical phase of the national lockdown.^20 38^ Although we performed hierarchical logistic regressions to examine departure from consistent patterns of association between the explanatory variables and physical activity, we could not formally test whether the proportional odds assumption for models with ordinal outcome variables was violated. However, analyses with the binary physical activity outcomes, as well as the relaxed proportional odds models (available in the online supplemental materials), suggested a similar pattern of results to the multilevel ordinal logistic proportional odds models.

This study relied on self-report measures, which are prone to recall and social desirability biases. In particular, the ordinal categorisation of physical activity obscures small fluctuations within categories over time, whereas fluctuations between categories are reported as behavioural changes.^25^ Future research may therefore benefit from using a range of subjective and objective measures, to enable a more nuanced understanding of older adults’ physical activity behaviour.^39 40^ Moreover, while we controlled for numerous covariates, the influence of unmeasured or residual confounding variables cannot be dismissed. We treated socioeconomic factors and covariates as time-constant variables. Although this is common practice, it is plausible that participants’ income or occupational position fluctuated during the COVID-19 pandemic.^33^

ELSA participants were predominantly white, limiting generalisability to other populations. We found preliminary evidence that non-white participants displayed lower physical activity levels than white older adults. Nevertheless, research with minority ethnic and racial groups is necessary to replicate these findings.^37^ This is particularly important given that ethnic minority groups in the UK have been disproportionately affected by the COVID-19 pandemic.^41^ Furthermore, as years of educational attainment (which would have allowed the inclusion of older adults with foreign qualifications) were not available in ELSA, data relating to participants’ highest educational qualifications were used. While the three-class ordinal National Statistics Socio-Economic Classification necessarily excludes participants who had never worked and were long-term unemployed from analyses, which could have affected our results, it is worth noting this response only applied to 0.5% of our sample.

Our findings have several implications. Notably, the results inform clinicians, policymakers and practitioners about socioeconomic subgroups that require targeting via intervention. In the context of restrictions on social contact, remotely delivered physical activity interventions may be an area of future research.^20^ A recent analysis showed that the magnitude of change in adults’ physical activity levels during the COVID-19 pandemic in England differed across activity modalities and demographic groups.^21^ Therefore, studies should explore whether these findings are applicable to older adults of varying socioeconomic status. Given the sustained influence of the COVID-19 pandemic on individuals’ lives and behaviours, it will be essential to develop policies and health promotion strategies to support older adults to perform physical activity at home or in a limited space.^42 43^ Moreover, the results reiterate the need for further work eliciting the mechanisms underlying associations between socioeconomic status and physical activity. In a qualitative study, health limitations, neighbourhood safety and lack of knowledge of physical activity guidelines were cited as the most prominent barriers among a subsample of older adults of low socioeconomic status in the UK.^14^ As such, identifying modifiable psychosocial and environmental factors differentially associated with physical activity behaviour in older adults of varying socioeconomic status, during and beyond the COVID-19 pandemic, should be a research priority.

Overall, the current study provides evidence for socioeconomic disparities in the physical activity levels of older adults in England, which largely persisted during the COVID-19 pandemic. Our findings emphasise the need to instate policies for the provision of targeted interventions to support physical activity in older adults, considering differences across socioeconomic groups in their design and implementation. Future research should replicate these findings over a longer follow-up period in cohorts with varying ethnic and sociocultural backgrounds to improve generalisability.

## supplementary material

10.1136/bmjph-2023-000100 online supplemental file 1

## Data Availability

Data are available in a public, open access repository.
