# Supplementary material for: Socioeconomic inequalities in physical activity among older adults before and during the COVID-19 pandemic: evidence from the English Longitudinal Study of Ageing
Source: BMJ Public Health. 2023 Sep 21;1(1):e000100. doi: 10.1136/bmjph-2023-000100 (PMC11812711; doi:10.1136/bmjph-2023-000100)
Supplement: online supplemental file 1 [file bmjph-1-1-s001.pdf]

SUPPLEMENTAL MATERIAL

Supplemental Appendix 1. STROBE checklist

STROBE Statement—checklist of items that should be included in reports of observational studies

	Item No	Recommendation
**Title and abstract**	1	(*a*) Indicate the study's design with a commonly used term in the title or the abstract **(page 2)**
		(*b*) Provide in the abstract an informative and balanced summary of what was done and what was found **(pages 2–3)**
**Introduction**		
Background/rationale	2	Explain the scientific background and rationale for the investigation being reported **(pages 5–7)**
Objectives	3	State specific objectives, including any prespecified hypotheses **(page 7)**
**Methods**		
Study design	4	Present key elements of study design early in the paper **(pages 7–8)**
Setting	5	Describe the setting, locations, and relevant dates, including periods of recruitment, exposure, follow-up, and data collection **(pages 7–8)**
Participants	6	(*a*) *Cohort study*—Give the eligibility criteria, and the sources and methods of selection of participants. Describe methods of follow-up **(pages 7–8 and 10–11)**<br>*Case-control study*—Give the eligibility criteria, and the sources and methods of case ascertainment and control selection. Give the rationale for the choice of cases and controls<br>*Cross-sectional study*—Give the eligibility criteria, and the sources and methods of selection of participants
		(*b*) *Cohort study*—For matched studies, give matching criteria and number of exposed and unexposed **N/A**<br>*Case-control study*—For matched studies, give matching criteria and the number of controls per case
Variables	7	Clearly define all outcomes, exposures, predictors, potential confounders, and effect modifiers. Give diagnostic criteria, if applicable **(pages 8–10)**
Data sources/ measurement	8*	For each variable of interest, give sources of data and details of methods of assessment (measurement). Describe comparability of assessment methods if there is more than one group **(pages 8–10)**
Bias	9	Describe any efforts to address potential sources of bias **(pages 10–12)**
Study size	10	Explain how the study size was arrived at **(pages 10–11)**
Quantitative variables	11	Explain how quantitative variables were handled in the analyses. If applicable, describe which groupings were chosen and why **(pages 8–12)**
Statistical methods	12	(*a*) Describe all statistical methods, including those used to control for confounding **(pages 10–12)**
		(*b*) Describe any methods used to examine subgroups and interactions **(pages 10–12)**
		(*c*) Explain how missing data were addressed **(pages 10–11)**
		(*d*) *Cohort study*—If applicable, explain how loss to follow-up was addressed **(pages 10–11)**<br>*Case-control study*—If applicable, explain how matching of cases and controls was addressed<br>*Cross-sectional study*—If applicable, describe analytical methods taking account of sampling strategy
		(*e*) Describe any sensitivity analyses **(pages 11–12)**

Continued on next page

**Results**		
Participants	13*	(a) Report numbers of individuals at each stage of study—eg numbers potentially eligible, examined for eligibility, confirmed eligible, included in the study, completing follow-up, and analysed **(Supplemental Material page 3)**
		(b) Give reasons for non-participation at each stage **(Supplemental Material page 3)**
		(c) Consider use of a flow diagram **(Supplemental Material page 3)**
Descriptive data	14*	(a) Give characteristics of study participants (eg demographic, clinical, social) and information on exposures and potential confounders **(pages 12–13)**
		(b) Indicate number of participants with missing data for each variable of interest **(Supplemental Material page 9)**
		(c) *Cohort study*—Summarise follow-up time (eg, average and total amount) **(Supplemental Material page 3)**
Outcome data	15*	*Cohort study*—Report numbers of outcome events or summary measures over time **(page 12 and Supplemental Material page 12)**
		Case-control study—Report numbers in each exposure category, or summary measures of exposure
		Cross-sectional study—Report numbers of outcome events or summary measures
Main results	16	(*a*) Give unadjusted estimates and, if applicable, confounder-adjusted estimates and their precision (eg, 95% confidence interval). Make clear which confounders were adjusted for and why they were included **(pages 9–10 and 13–14 and Supplemental Material page 10)**
		(*b*) Report category boundaries when continuous variables were categorized **(page 9)**
		(*c*) If relevant, consider translating estimates of relative risk into absolute risk for a meaningful time period **N/A**
Other analyses	17	Report other analyses done—eg analyses of subgroups and interactions, and sensitivity analyses **(pages 11, 13–15 and Supplemental Material pages 4–8, and 11–13)**
**Discussion**		
Key results	18	Summarise key results with reference to study objectives **(pages 15–16)**
Limitations	19	Discuss limitations of the study, taking into account sources of potential bias or imprecision. Discuss both direction and magnitude of any potential bias **(pages 17–18)**
Interpretation	20	Give a cautious overall interpretation of results considering objectives, limitations, multiplicity of analyses, results from similar studies, and other relevant evidence **(pages 15–19)**
Generalisability	21	Discuss the generalisability (external validity) of the study results **(pages 16–19)**
**Other information**		
Funding	22	Give the source of funding and the role of the funders for the present study and, if applicable, for the original study on which the present article is based **(page 20)**

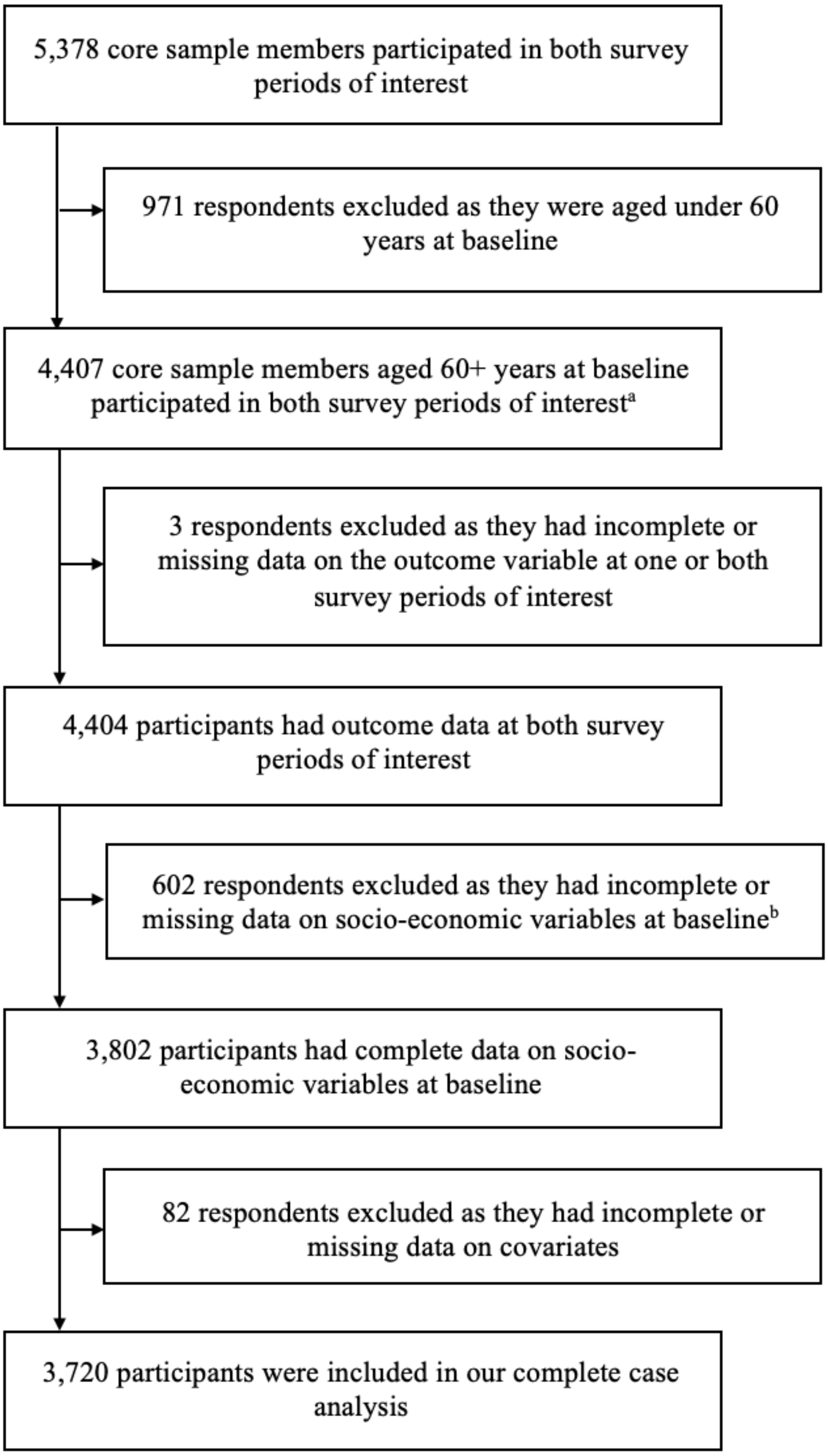

Supplemental Figure S1. Flow of study members into the complete case analytical sample.

[a]Sample for multiple imputation analyses.
[b]Includes extended missing values.
Note: Covariates were biological sex, age, ethnicity, living status, the presence of any limiting long-standing illness, disability, or infirmity, depressive symptoms, and shielding during the COVID-19 pandemic.

Supplemental Table S1. Multilevel logistic models of physical activity at pre- and intra-pandemic across socio-economic groups (unadjusted)

	Model 1[a]	Model 1[b]	Model 2[a]	Model 2[b]	Model 3[a]	Model 3[b]	Model 4[a]	Model 4[b]
**Fixed effects**								
Time								
Baseline (ref.)	1.00	1.00	1.00	1.00	1.00	1.00	1.00	1.00
During COVID-19	0.73 (0.43, 1.23)	1.08 (0.74, 1.57)	0.59 (0.39, 0.89)**	0.95 (0.72, 1.25)	0.70 (0.43, 1.14)	0.78 (0.52, 1.17)	0.90 (0.46, 1.76)	1.03 (0.63, 1.69)
Education								
Low (ref.)	1.00	1.00					1.00	1.00
Medium	3.01 (1.78, 5.10)***	2.74 (1.80, 4.16)***					2.14 (1.26, 3.63)**	1.86 (1.21, 2.84)**
High	5.16 (3.05, 8.73)***	10.52 (6.90, 16.04)***					2.37 (1.33, 4.20)**	4.52 (2.88, 7.11)***
Occupational class								
Routine and manual (ref.)			1.00	1.00			1.00	1.00
Intermediate			2.29 (1.35, 3.88)**	3.43 (2.40, 4.92)***			1.40 (0.81, 2.43)	1.93 (1.33, 2.80)***
Higher			3.75 (2.28, 6.15)***	5.53 (3.94, 7.78)***			1.66 (0.92, 3.00)	1.69 (1.17, 2.44)**
Wealth								
1st quintile (ref.)					1.00	1.00	1.00	1.00
2nd quintile					2.78 (1.59, 4.84)***	2.72 (1.72, 4.31)***	2.28 (1.31, 3.97)**	2.05 (1.28, 3.27)**
3rd quintile					5.15 (2.72, 9.74)***	3.67 (2.32, 5.81)***	3.89 (2.07, 7.31)***	2.39 (1.50, 3.83)***
4th quintile					5.02 (2.64, 9.56)***	7.56 (4.69, 12.18)***	3.30 (1.69, 6.42)***	4.07 (2.49, 6.65)***
5th quintile					8.25 (4.15, 16.39)***	13.87 (8.52, 22.57)***	4.82 (2.38, 9.75)***	5.82 (3.54, 9.57)***
Education × Time[c]								
Medium vs low	0.57 (0.30, 1.06)	0.81 (0.51, 1.28)					0.62 (0.33, 1.16)	0.82 (0.51, 1.31)
High vs low	0.55 (0.29, 1.05)	0.61 (0.40, 0.94)*					0.73 (0.37, 1.47)	0.63 (0.38, 1.02)
Occupational class × Time[c]								
Intermediate vs routine and manual			0.90 (0.51, 1.59)	0.84 (0.56, 1.27)			1.04 (0.58, 1.86)	0.86 (0.56, 1.32)
Higher vs routine and manual			0.57 (0.32, 1.02)	0.73 (0.51, 1.03)			0.70 (0.36, 1.34)	0.81 (0.54, 1.23)
Wealth × Time[c]								
2nd quintile vs 1st quintile					0.55 (0.29, 1.06)	1.12 (0.65, 1.91)	0.60 (0.31, 1.15)	1.21 (0.70, 2.09)
3rd quintile vs 1st quintile					0.52 (0.24, 1.09)	0.99 (0.59, 1.66)	0.58 (0.27, 1.23)	1.12 (0.66, 1.91)
4th quintile vs 1st quintile					0.75 (0.35, 1.60)	0.94 (0.56, 1.58)	0.90 (0.41, 1.96)	1.12 (0.65, 1.94)
5th quintile vs 1st quintile					0.54 (0.26, 1.10)	1.09 (0.66, 1.80)	0.67 (0.31, 1.44)	1.40 (0.82, 2.41)
**Random effects**								
Variance intercept	3.14 (2.06, 4.78)	5.64 (4.56, 6.98)	3.15 (2.06, 4.82)	5.88 (4.77, 7.26)	2.91 (1.91, 4.44)	5.32 (4.32, 6.57)	2.93 (1.92, 4.45)	5.02 (4.07, 6.20)

Data are odds ratios and 95 % confidence intervals. *ref.*, reference category. $^{*}p \le 0.05$, $^{**}p \le 0.01$, $^{***}p \le 0.001$.
Number of participants = 3,720 (Level 2); number of observations = 7,440 (Level 1).
[a]Outcome is inactive (coded as 0) versus mild, moderate, or vigorous physical activity (coded as 1).
[b]Outcome is inactive, mild physical activity, or moderate physical activity (coded as 0) versus vigorous physical activity (coded as 1).
[c]Interaction terms.

Supplemental Table S2. Multilevel logistic models of physical activity at pre- and intra-pandemic across socio-economic groups, adjusted for covariates

	Model 1[a]	Model 1[b]	Model 2[a]	Model 2[b]	Model 3[a]	Model 3[b]	Model 4[a]	Model 4[b]
**Fixed effects**								
Time								
Baseline (ref.)	1.00	1.00	1.00	1.00	1.00	1.00	1.00	1.00
During COVID-19	0.72 (0.42, 1.24)	1.08 (0.74, 1.59)	0.59 (0.40, 0.89)**	0.95 (0.72, 1.26)	0.70 (0.43, 1.14)	0.78 (0.52, 1.17)	0.88 (0.46, 1.71)	1.03 (0.62, 1.71)
Education								
Low (ref.)	1.00	1.00					1.00	1.00
Medium	1.74 (1.06, 2.85)*	1.59 (1.05, 2.41)*					1.40 (0.85, 2.32)	1.23 (0.81, 1.87)
High	2.59 (1.55, 4.30)***	4.66 (3.09, 7.04)***					1.51 (0.85, 2.67)	2.66 (1.72, 4.11)***
Occupational class								
Routine and manual (ref.)			1.00	1.00			1.00	1.00
Intermediate			1.51 (0.94, 2.43)	2.55 (1.81, 3.61)***			1.18 (0.71, 1.95)	1.85 (1.30, 2.65)***
Higher			2.55 (1.62, 4.02)***	3.50 (2.54, 4.82)***			1.71 (0.99, 2.96)*	1.70 (1.19, 2.42)**
Wealth								
1st quintile (ref.)					1.00	1.00	1.00	1.00
2nd quintile					1.96 (1.17, 3.30)**	1.92 (1.22, 3.01)**	1.70 (1.01, 2.86)*	1.59 (1.00, 2.50)*
3rd quintile					2.79 (1.54, 5.05)***	2.12 (1.36, 3.29)***	2.39 (1.31, 4.33)**	1.55 (0.98, 2.43)
4th quintile					2.32 (1.28, 4.20)**	3.41 (2.16, 5.39)***	1.73 (0.93, 3.21)	2.20 (1.38, 3.52)***
5th quintile					3.71 (1.90, 7.24)***	6.07 (3.83, 9.62)***	2.54 (1.28, 5.04)**	3.17 (1.97, 5.11)***
Education × Time[c]								
Medium vs low	0.58 (0.31, 1.08)	0.81 (0.51, 1.29)					0.65 (0.34, 1.21)	0.82 (0.51, 1.32)
High vs low	0.56 (0.30, 1.08)	0.62 (0.40, 0.95)*					0.75 (0.37, 1.53)	0.63 (0.39, 1.03)
Occupational class × Time[c]								
Intermediate vs routine and manual			0.90 (0.51, 1.58)	0.85 (0.56, 1.28)			1.04 (0.58, 1.86)	0.86 (0.56, 1.32)
Higher vs routine and manual			0.57 (0.32, 1.01)	0.73 (0.51, 1.03)			0.69 (0.36, 1.33)	0.81 (0.54, 1.22)
Wealth × Time[c]								
2nd quintile vs 1st quintile					0.55 (0.29, 1.06)	1.12 (0.65, 1.92)	0.62 (0.32, 1.18)	1.20 (0.69, 2.07)
3rd quintile vs 1st quintile					0.53 (0.25, 1.11)	1.00 (0.59, 1.67)	0.58 (0.28, 1.23)	1.12 (0.66, 1.91)
4th quintile vs 1st quintile					0.74 (0.35, 1.59)	0.95 (0.56, 1.59)	0.90 (0.41, 1.98)	1.12 (0.65, 1.94)
5th quintile vs 1st quintile					0.54 (0.26, 1.11)	1.10 (0.66, 1.81)	0.68 (0.32, 1.47)	1.40 (0.82, 2.40)
Biological sex								
Male (ref.)	1.00	1.00	1.00	1.00	1.00	1.00	1.00	1.00
Female	1.87 (1.43, 2.44)***	0.73 (0.59, 0.92)**	1.80 (1.37, 2.35)***	0.64 (0.52, 0.80)***	1.76 (1.35, 2.29)***	0.64 (0.52, 0.80)***	1.83 (1.40, 2.39)***	0.70 (0.56, 0.87)**
Age								
60-69 years (ref.)	1.00	1.00	1.00	1.00	1.00	1.00	1.00	1.00
70-79 years	1.01 (0.75, 1.36)	0.63 (0.50, 0.79)***	0.99 (0.74, 1.33)	0.60 (0.47, 0.75)***	0.95 (0.71, 1.28)	0.57 (0.45, 0.71)***	0.97 (0.72, 1.30)	0.60 (0.48, 0.75)***

80+ years	0.41 (0.29, 0.58)***	0.19 (0.13, 0.27)***	0.38 (0.27, 0.54)***	0.16 (0.11, 0.23)***	0.36 (0.26, 0.51)***	0.16 (0.11, 0.23)***	0.38 (0.27, 0.54)***	0.18 (0.12, 0.26)***
Ethnicity								
White (ref.)	1.00	1.00	1.00	1.00	1.00	1.00	1.00	1.00
non-White	0.69 (0.33, 1.42)	0.36 (0.19, 0.71)**	0.71 (0.34, 1.47)	0.40 (0.20, 0.78)**	0.72 (0.35, 1.49)	0.41 (0.21, 0.79)**	0.73 (0.35, 1.53)	0.39 (0.20, 0.76)**
Living status								
Living alone (ref.)	1.00	1.00	1.00	1.00	1.00	1.00	1.00	1.00
Not living alone	1.04 (0.76, 1.42)	1.43 (1.10, 1.86)**	1.04 (0.76, 1.43)	1.46 (1.12, 1.90)**	0.91 (0.67, 1.25)	1.06 (0.82, 1.39)	0.94 (0.68, 1.29)	1.14 (0.88, 1.48)
Limiting long-standing illness, disability, or infirmity								
No (ref.)	1.00	1.00	1.00	1.00	1.00	1.00	1.00	1.00
Yes	0.25 (0.19, 0.34)***	0.27 (0.21, 0.35)***	0.25 (0.19, 0.34)***	0.27 (0.21, 0.35)***	0.27 (0.20, 0.36)***	0.31 (0.24, 0.39)***	0.27 (0.20, 0.36)***	0.31 (0.24, 0.40)***
Depressive symptoms	0.86 (0.80, 0.91)***	0.83 (0.77, 0.89)***	0.86 (0.80, 0.91)***	0.83 (0.77, 0.90)***	0.87 (0.81, 0.93)***	0.85 (0.78, 0.91)***	0.87 (0.82, 0.93)***	0.85 (0.79, 0.92)***
Shielding								
No (ref.)	1.00	1.00	1.00	1.00	1.00	1.00	1.00	1.00
Yes	0.39 (0.28, 0.55)***	0.47 (0.31, 0.69)***	0.39 (0.28, 0.54)***	0.45 (0.30, 0.67)***	0.40 (0.28, 0.56)***	0.49 (0.33, 0.73)***	0.40 (0.28, 0.56)***	0.51 (0.34, 0.76)***
**Random effects**								
Variance intercept	1.62 (0.99, 2.65)	4.12 (3.34, 5.08)	1.58 (0.96, 2.60)	4.17 (3.39, 5.14)	1.56 (0.94, 2.59)	4.00 (3.24, 4.93)	1.61 (0.98, 2.63)	3.87 (3.13, 4.79)

Data are odds ratios and 95 % confidence intervals. *ref.*, reference category. $^{*}p \leq 0.05$, $^{**}p \leq 0.01$, $^{***}p \leq 0.001$.
Number of participants = 3,720 (Level 2); number of observations = 7,440 (Level 1).
[a]Outcome is inactive (coded as 0) versus mild, moderate, or vigorous physical activity (coded as 1).
[b]Outcome is inactive, mild physical activity, or moderate physical activity (coded as 0) versus vigorous physical activity (coded as 1).
[c]Interaction terms.

Supplemental Table S3. Two-level ordinal logistic regression models (full proportional odds) of physical activity at pre- and intra-pandemic across socio-economic groups (unadjusted)

	Model 1	Model 2	Model 3
**Fixed effects**			
Time	-0.05 (0.14)	-0.12 (0.10)	-0.14 (0.14)
Education (reference: Low)			
Medium	1.06 (0.16)***		
High	2.09 (0.16)***		
Occupational class (reference: Routine and manual)			
Intermediate		1.03 (0.15)***	
Higher		1.51 (0.13)***	
Wealth (reference: 1st quintile)			
2nd quintile			0.99 (0.17)***
3rd quintile			1.40 (0.17)***
4th quintile			2.05 (0.18)***
5th quintile			2.50 (0.19)***
Education × Time[a]			
Medium vs low	-0.28 (0.16)		
High vs low	-0.44 (0.16)**		
Occupational class × Time[a]			
Intermediate vs routine and manual		-0.28 (0.15)	
Higher vs routine and manual		-0.41 (0.13)**	
Wealth × Time[a]			
2nd quintile vs 1st quintile			-0.24 (0.18)
3rd quintile vs 1st quintile			-0.22 (0.18)
4th quintile vs 1st quintile			-0.33 (0.19)
5th quintile vs 1st quintile			-0.18 (0.18)
**Random effects**			
Variance (Level 2)	4.13	4.36	3.92

Data are estimates and standard errors. All values are weighted estimates. *$p \leq 0.05$, **$p \leq 0.01$, ***$p \leq 0.001$.
Number of participants = 3,720 (Level 2); number of observations = 7,440 (Level 1).
[a]Interaction terms.

Supplemental Table S4. Two-level ordinal logistic regression models (relaxed proportional odds) of physical activity at pre- and intra-pandemic across socio-economic groups (unadjusted)

	Model 1	Model 2	Model 3
**Fixed effects**			
†Time	-0.06 (0.14)	-0.12 (0.10)	-0.14 (0.14)
†Education × Time[a]			
Medium vs low	-0.28 (0.17)		
High vs low	-0.42 (0.16)**		
†Occupational class × Time[a]			
Intermediate vs routine and manual		-0.27 (0.15)	
Higher vs routine and manual		-0.40 (0.13)**	
†Wealth × Time[a]			
2nd quintile vs 1st quintile			-0.24 (0.18)
3rd quintile vs 1st quintile			-0.23 (0.19)
4th quintile vs 1st quintile			-0.33 (0.19)
5th quintile vs 1st quintile			-0.17 (0.18)
**Intercept 1**			
Education (reference: Low)			
Medium	-0.90 (0.21)***		
High	-1.63 (0.22)***		
Occupational class (reference: Routine and manual)			
Intermediate		-0.95 (0.22)***	
Higher		-1.33 (0.19)***	
Wealth (reference: 1st quintile)			
2nd quintile			-0.89 (0.23)***
3rd quintile			-1.43 (0.24)***
4th quintile			-1.77 (0.26)***
5th quintile			-2.06 (0.28)***
**Intercept 2**			
Education (reference: Low)			
Medium	-1.24 (0.18)***		
High	-2.16 (0.18)***		
Occupational class (reference: Routine and manual)			
Intermediate		-0.88 (0.17)***	
Higher		-1.46 (0.16)***	
Wealth (reference: 1st quintile)			
2nd quintile			-0.97 (0.19)***
3rd quintile			-1.52 (0.20)***
4th quintile			-2.25 (0.21)***
5th quintile			-2.59 (0.22)***
**Intercept 3**			
Education (reference: Low)			
Medium	-0.98 (0.19)***		
High	-2.12 (0.18)***		
Occupational class (reference: Routine and manual)			
Intermediate		-1.18 (0.16)***	
Higher		-1.61 (0.15)***	
Wealth (reference: 1st quintile)			
2nd quintile			-1.05 (0.20)***
3rd quintile			-1.32 (0.21)***
4th quintile			-1.98 (0.21)***
5th quintile			-2.49 (0.21)***
**Random effects**			
Variance (Level 2)	4.10	4.37	3.92

Data are estimates and standard errors. All values are weighted estimates. *$p \leq 0.05$, **$p \leq 0.01$, ***$p \leq 0.001$.
Number of participants = 3,720 (Level 2); number of observations = 7,440 (Level 1).
†Full proportional odds.
[a]Interaction terms.

Supplemental Table S5. Number of observations imputed per dataset

Variable	Complete	Incomplete	Imputed	Total
**Overall**				
Physical activity (baseline)	4,406	1	1	4,407
Physical activity (follow-up)	4,405	2	2	4,407
Education	4,069	2	2	4,071 (336 extended missing values
Occupational class	4,157	228	213	4,385 (22 extended missing values)
Wealth	4,368	39	37	4,407
Biological sex	4,407	0	0	4,407
Age	4,407	0	0	4,407
Ethnicity	4,407	0	0	4,407
Living status	4,407	0	0	4,407
Limiting long-standing illness, disability, or infirmity	4,403	4	4	4,407
Depressive symptoms	4,298	109	101	4,407
Shielding	4,405	2	2	4,407
Self-reported general health[a]	4,331	76	71	4,407
Alcohol consumption[a]	4,112	295	269	4,407
Smoking status[a]	4,407	0	0	4,407

[a]Auxiliary variables included in the imputation model.

Note: Due to extended missing values for education and/or occupational class, some missing imputed values were produced for the other variables.

Supplemental Table S6. Multilevel ordered logistic model of physical activity at pre- and intra-pandemic across socio-economic groups (unadjusted)

	Model 1	Model 2	Model 3	Model 4
**Fixed effects**				
Time				
Baseline (reference)	1.00	1.00	1.00	1.00
During COVID-19	0.95 (0.73, 1.23)	0.89 (0.73, 1.08)	0.87 (0.66, 1.14)	1.08 (0.77, 1.52)
Education				
Low (reference)	1.00			1.00
Medium	2.87 (2.11, 3.90)***			1.96 (1.45, 2.65)***
High	8.09 (5.92, 11.04)***			3.56 (2.55, 4.97)***
Occupational class				
Routine and manual (reference)		1.00		1.00
Intermediate		2.80 (2.10, 3.74)***		1.55 (1.16, 2.08)**
Higher		4.52 (3.48, 5.88)***		1.51 (1.13, 2.02)**
Wealth				
1st quintile (reference)			1.00	1.00
2nd quintile			2.70 (1.91, 3.79)***	2.07 (1.48, 2.89)***
3rd quintile			4.06 (2.88, 5.71)***	2.80 (2.01, 3.91)***
4th quintile			7.73 (5.41, 11.04)***	4.44 (3.09, 6.38)***
5th quintile			12.18 (8.43, 17.59)***	5.93 (4.09, 8.60)***
Education × Time[a]				
Medium vs low	0.76 (0.55, 1.04)			0.82 (0.59, 1.13)
High vs low	0.64 (0.47, 0.88)**			0.74 (0.52, 1.06)
Occupational class × Time[a]				
Intermediate vs routine and manual		0.76 (0.57, 1.02)		0.81 (0.60, 1.10)
Higher vs routine and manual		0.67 (0.51, 0.86)**		0.75 (0.55, 1.01)
Wealth × Time[a]				
2nd quintile vs 1st quintile			0.79 (0.55, 1.12)	0.86 (0.60, 1.23)
3rd quintile vs 1st quintile			0.80 (0.56, 1.15)	0.91 (0.63, 1.31)
4th quintile vs 1st quintile			0.72 (0.49, 1.04)	0.87 (0.59, 1.30)
5th quintile vs 1st quintile			0.83 (0.58, 1.18)	1.06 (0.72, 1.56)
**Random effects**				
Variance intercept	4.11 (3.57, 4.74)	4.34 (3.79, 4.99)	3.91 (3.39, 4.51)	3.72 (3.21, 4.31)

Data are odds ratios and 95 % confidence intervals. All values are weighted estimates. *$p \leq 0.05$, **$p \leq 0.01$, ***$p \leq 0.001$.
Number of participants = 3,720 (Level 2); number of observations = 7,440 (Level 1).
[a]Interaction terms.

Supplemental Table S7. Multilevel ordered logistic model of physical activity at pre- and intra-pandemic across socio-economic groups using multiple imputation for missing data (unadjusted)

	Model 1[a]	Model 2[b]	Model 3[c]	Model 4[d]
**Fixed effects**				
Time				
Baseline (reference)	1.00	1.00	1.00	1.00
During COVID-19	0.96 (0.73, 1.26)	0.95 (0.78, 1.15)	0.99 (0.77, 1.28)	1.03 (0.74, 1.44)
Education				
Low (reference)	1.00			1.00
Medium	2.83 (2.05, 3.90)***			1.80 (1.32, 2.45)***
High	8.19 (5.88, 11.40)***			3.52 (2.49, 4.98)***
Occupational class				
Routine and manual (reference)		1.00		1.00
Intermediate		2.59 (1.92, 3.50)***		1.51 (1.09, 2.07)**
Higher		4.50 (3.41, 5.94)***		1.49 (1.08, 2.05)*
Wealth				
1st quintile (reference)			1.00	1.00
2nd quintile			3.12 (2.26, 4.30)***	2.14 (1.55, 2.96)***
3rd quintile			4.35 (3.11, 6.10)***	2.91 (2.07, 4.08)***
4th quintile			8.63 (6.06, 12.27)***	4.71 (3.27, 6.79)***
5th quintile			11.89 (8.25, 17.15)***	5.24 (3.57, 7.70)***
Education × Time[e]				
Medium vs low	0.77 (0.56, 1.06)			0.84 (0.61, 1.16)
High vs low	0.68 (0.49, 0.92)**			0.75 (0.53, 1.07)
Occupational class × Time[e]				
Intermediate vs routine and manual		0.75 (0.55, 1.00)*		0.84 (0.61, 1.17)
Higher vs routine and manual		0.69 (0.53, 0.89)**		0.78 (0.57, 1.07)
Wealth × Time[e]				
2nd quintile vs 1st quintile			0.73 (0.52, 1.02)	0.88 (0.62, 1.24)
3rd quintile vs 1st quintile			0.71 (0.51, 1.00)*	0.89 (0.62, 1.26)
4th quintile vs 1st quintile			0.64 (0.45, 0.92)*	0.91 (0.62, 1.34)
5th quintile vs 1st quintile			0.83 (0.59, 1.18)	1.24 (0.84, 1.85)
**Random effects**				
Variance intercept	4.08 (3.51, 4.74)	4.18 (3.62, 4.83)	3.78 (3.26, 4.39)	3.67 (3.15, 4.28)

Data are odds ratios and 95 % confidence intervals. All values are weighted estimates. $*p \leq 0.05$, $**p \leq 0.01$, $***p \leq 0.001$.
[a]Number of participants = 4,071 (Level 2); number of observations = 8,142 (Level 1).
[b]Number of participants = 4,370 (Level 2); number of observations = 8,740 (Level 1).
[c]Number of participants = 4,405 (Level 2); number of observations = 8,810 (Level 1).
[d]Number of participants = 4,054 (Level 2); number of observations = 8,108 (Level 1).
[e]Interaction terms.
Note: The "cmdok" option was used to force the "meologit" command to run on imputed data.

Supplemental Table S8. Predictive margins probabilities of the ordinal physical activity outcome adjusted for each combination of the socio-economic and time variables

	Inactive	Mild activity	Moderate activity	Vigorous activity
	**Margin (95 % CI)**	**Margin (95 % CI)**	**Margin (95 % CI)**	**Margin (95 % CI)**
**Education**[a]				
Low				
Baseline (reference)	7.92 (6.24, 9.61)	18.32 (16.05, 20.60)	58.46 (56.36, 60.57)	15.29 (12.32, 18.27)
During COVID-19	8.22 (6.53, 9.91)	18.73 (16.27, 21.19)	58.32 (56.20, 60.44)	14.73 (11.67, 17.79)
Medium				
Baseline (reference)	5.62 (4.55, 6.70)	14.79 (13.20, 16.39)	58.58 (56.47, 60.69)	21.01 (18.54, 23.47)
During COVID-19	7.30 (5.93, 8.67)	17.43 (15.60, 19.26)	58.69 (56.60, 60.78)	16.58 (14.21, 18.94)
High				
Baseline (reference)	2.79 (2.14, 3.44)	9.13 (7.86, 10.40)	53.30 (51.03, 55.56)	34.79 (31.84, 37.73)
During COVID-19	4.27 (3.32, 5.22)	12.33 (10.80, 13.86)	57.26 (55.09, 59.42)	26.14 (23.29, 29.00)
**Occupational class**[b]				
Routine and manual				
Baseline (reference)	7.41 (6.09, 8.74)	17.25 (15.50, 19.00)	58.25 (56.18, 60.31)	17.09 (14.72, 19.46)
During COVID-19	8.09 (6.67, 9.50)	18.19 (16.29, 20.08)	58.04 (55.98, 60.11)	15.68 (13.31, 18.06)
Intermediate				
Baseline (reference)	4.43 (3.39, 5.47)	12.38 (10.70, 14.05)	56.68 (54.46, 58.91)	26.51 (23.23, 29.80)
During COVID-19	6.04 (4.72, 7.36)	15.18 (13.25, 17.11)	58.15 (56.06, 60.25)	20.63 (17.53, 23.73)
Higher				
Baseline (reference)	3.21 (2.50, 3.93)	9.91 (8.59, 11.24)	53.84 (51.55, 56.13)	33.03 (30.05, 36.02)
During COVID-19	4.95 (3.89, 6.02)	13.34 (11.70, 14.98)	57.38 (55.21, 59.56)	24.32 (21.32, 27.32)
**Wealth**[c]				
1st quintile				
Baseline (reference)	8.46 (6.66, 10.26)	19.51 (17.11, 21.91)	58.52 (56.28, 60.75)	13.52 (10.78, 16.25)
During COVID-19	9.34 (7.44, 11.24)	20.67 (18.10, 23.23)	57.82 (55.43, 60.20)	12.17 (9.57, 14.77)
2nd quintile				
Baseline (reference)	5.21 (4.05, 6.37)	14.39 (12.47, 16.30)	59.17 (57.00, 61.33)	21.24 (18.03, 24.45)
During COVID-19	7.12 (5.55, 8.69)	17.57 (15.28, 19.86)	59.26 (57.12, 61.39)	16.05 (13.10, 19.00)
3rd quintile				
Baseline (reference)	4.44 (3.44, 5.45)	12.93 (11.14, 14.72)	58.49 (56.22, 60.76)	24.14 (20.78, 27.49)
During COVID-19	6.00 (4.61, 7.39)	15.78 (13.67, 17.89)	59.42 (57.32, 61.52)	18.80 (15.60, 21.99)
4th quintile				
Baseline (reference)	3.20 (2.34, 4.06)	10.28 (8.59, 11.97)	56.00 (53.49, 58.50)	30.52 (26.65, 34.39)
During COVID-19	4.74 (3.48, 6.00)	13.51 (11.35, 15.67)	58.81 (56.54, 61.09)	22.94 (18.92, 26.96)
5th quintile				
Baseline (reference)	2.13 (1.50, 2.76)	7.58 (6.09, 9.07)	51.28 (48.26, 54.31)	39.01 (34.58, 43.43)
During COVID-19	2.83 (2.00, 3.65)	9.39 (7.63, 11.15)	54.72 (51.89, 57.55)	33.06 (28.58, 37.54)

CI, confidence intervals. Data are reported as percentages. All predicted values were computed using the "predict (mu fixedonly)" option to fix the random effects of the respective multilevel ordered logistic model to zero, the "asobserved" option for covariates, and the "vce(unconditional)" option.
[a]Calculated after Model 1 with covariates (complete case sample).
[b]Calculated after Model 2 with covariates (complete case sample).
[c]Calculated after Model 3 with covariates (complete case sample).

Supplemental Table S9. Multilevel ordered logistic model of physical activity at pre- and intra-pandemic across socio-economic groups using multiple imputation for missing data, adjusted for covariates

	Model 1[a]	Model 2[b]	Model 3[c]	Model 4[d]
**Fixed effects**				
Time				
Baseline (reference)	1.00	1.00	1.00	1.00
During COVID-19	0.97 (0.74, 1.27)	0.95 (0.78, 1.15)	0.99 (0.76, 1.28)	1.05 (0.75, 1.46)
Education				
Low (reference)	1.00			1.00
Medium	1.62 (1.19, 2.20)**			1.21 (0.90, 1.64)
High	3.74 (2.73, 5.12)***			2.16 (1.55, 3.02)***
Occupational class				
Routine and manual (reference)		1.00		1.00
Intermediate		1.87 (1.43, 2.45)***		1.39 (1.03, 1.86)*
Higher		2.77 (2.17, 3.55)***		1.48 (1.11, 1.98)**
Wealth				
1st quintile (reference)			1.00	1.00
2nd quintile			2.07 (1.56, 2.76)***	1.58 (1.18, 2.12)**
3rd quintile			2.56 (1.91, 3.44)***	1.84 (1.37, 2.49)***
4th quintile			3.67 (2.69, 5.01)***	2.39 (1.73, 3.32)***
5th quintile			4.97 (3.58, 6.89)***	2.71 (1.91, 3.83)***
Education × Time[e]				
Medium vs low	0.77 (0.55, 1.06)			0.83 (0.60, 1.15)
High vs low	0.67 (0.49, 0.91)**			0.74 (0.52, 1.05)
Occupational class × Time[e]				
Intermediate vs routine and manual		0.75 (0.56, 1.01)		0.85 (0.61, 1.18)
Higher vs routine and manual		0.69 (0.54, 0.90)**		0.79 (0.58, 1.08)
Wealth × Time[e]				
2nd quintile vs 1st quintile			0.72 (0.52, 1.00)*	0.87 (0.62, 1.22)
3rd quintile vs 1st quintile			0.71 (0.50, 1.00)*	0.88 (0.62, 1.25)
4th quintile vs 1st quintile			0.65 (0.46, 0.93)*	0.91 (0.62, 1.34)
5th quintile vs 1st quintile			0.83 (0.59, 1.18)	1.23 (0.83, 1.82)
Biological sex				
Male (reference)	1.00	1.00	1.00	1.00
Female	0.91 (0.75, 1.09)	0.82 (0.69, 0.97)*	0.79 (0.67, 0.93)**	0.90 (0.75, 1.07)
Age				
60-69 years (reference)	1.00	1.00	1.00	1.00
70-79 years	0.80 (0.67, 0.97)*	0.76 (0.63, 0.91)**	0.72 (0.61, 0.86)***	0.76 (0.63, 0.91)**
80+ years	0.31 (0.24, 0.41)***	0.26 (0.20, 0.33)***	0.25 (0.20, 0.32)***	0.28 (0.22, 0.37)***
Ethnicity				
White (reference)	1.00	1.00	1.00	1.00
non-White	0.64 (0.41, 1.02)	0.68 (0.44, 1.06)	0.73 (0.47, 1.14)	0.68 (0.42, 1.09)
Living status				
Living alone (reference)	1.00	1.00	1.00	1.00
Not living alone	1.31 (1.04, 1.65)*	1.26 (1.01, 1.58)*	0.99 (0.80, 1.22)	1.10 (0.88, 1.38)
Limiting long-standing illness, disability, or infirmity				
No (reference)	1.00	1.00	1.00	1.00
Yes	0.23 (0.18, 0.28)***	0.23 (0.19, 0.29)***	0.26 (0.21, 0.32)***	0.26 (0.21, 0.32)***
Depressive symptoms	0.85 (0.80, 0.90)***	0.85 (0.80, 0.90)***	0.86 (0.82, 0.91)***	0.88 (0.83, 0.93)***
Shielding				
No (reference)	1.00	1.00	1.00	1.00
Yes	0.42 (0.32, 0.55)***	0.43 (0.33, 0.55)***	0.44 (0.35, 0.57)***	0.47 (0.36, 0.61)***
**Random effects**				
Variance intercept	2.67 (2.26, 3.16)	2.61 (2.21, 3.08)	2.48 (2.10, 2.94)	2.54 (2.14, 3.01)

Data are odds ratios and 95 % confidence intervals. All values are weighted estimates. *$p \leq 0.05$, **$p \leq 0.01$, ***$p \leq 0.001$.
[a]Number of participants = 4,069 (Level 2); number of observations = 8,138 (Level 1).
[b]Number of participants = 4,365 (Level 2); number of observations = 8,730 (Level 1).
[c]Number of participants = 4,397 (Level 2); number of observations = 8,794 (Level 1).
[d]Number of participants = 4,054 (Level 2); number of observations = 8,108 (Level 1).
[e]Interaction terms.
Note: The "cmdok" option was used to force the "meologit" command to run on imputed data.
